# Checkpoint Inhibitor Pneumonitis: Key Insights for Pulmonologists

**DOI:** 10.3390/medicina61112064

**Published:** 2025-11-19

**Authors:** Candela Serra, Mariana Benegas, Xavier Alsina-Restoy, Nuria Roger-Casals, Fernanda Hernández-González

**Affiliations:** 1 Department of Pulmonary Medicine, Consorci Hospitalari de Vic, 08500 Barcelona, Spain; nroger@chv.cat; 2 Radiology Department, Hospital Clínic, 08036 Barcelona, Spain; mnbenega@clinic.cat; 3 Department of Pulmonary Medicine, Hospital Clínic, Respiratory Institute, 08036 Barcelona, Spain; xalsina@clinic.cat (X.A.-R.); fhernandez@clinic.cat (F.H.-G.)

**Keywords:** pneumonitis, immune checkpoint inhibitors, lung toxicity, pulmonologist

## Abstract

Immune checkpoint inhibitors (ICIs) have transformed cancer therapy, offering significant efficacy and a generally favorable safety profile. However, they are associated with a spectrum of immune-related adverse events (irAEs), among which pneumonitis stands out due to its relatively high mortality. This condition is characterized by nonspecific clinical manifestations and a broad differential diagnosis, often requiring the involvement of pulmonologists for accurate diagnosis and management. Given its potential severity, it is crucial for pulmonologists to be well-versed in recognizing and addressing checkpoint inhibitor pneumonitis (CIP). In this narrative review, we examine reported cases of pneumonitis in patients with various types of cancer treated with ICIs. The article explores the mechanisms of action of ICIs, the underlying pathophysiology of pneumonitis, associated risk factors, clinical presentation, diagnostic approaches, and current treatment strategies, intended to support pulmonologists in improving early detection and implementing evidence-based management of this potentially life-threatening complication.

## 1. Introduction

Immunotherapy has emerged as one of the most transformative advances in modern oncology, markedly improving survival outcomes across a wide range of malignancies. Among immunotherapeutic strategies, immune checkpoint inhibitors (ICIs) have gained prominence due to their ability to restore and potentiate the host immune response against tumor antigens. These agents function by blocking inhibitory pathways that normally restrain T-cell activation, thereby enabling cytotoxic T cells to mount an effective antitumor response.

However, by unleashing T-cell activity, ICIs can also disrupt immune tolerance to self-antigens, leading to a spectrum of immune-related adverse events (irAEs). These toxicities can affect nearly any organ system, with pulmonary involvement—specifically, checkpoint inhibitor pneumonitis (CIP)—representing one of the most serious and potentially fatal complications [1,2].

Despite its clinical relevance, CIP lacks a standardized diagnostic definition across literature. It is generally described as inflammation of the lung parenchyma, although some sources emphasize exclusion of infectious etiologies, while others highlight predominant interstitial and alveolar involvement [3].

Diagnosing CIP remains a challenge and requires a high index of suspicion, a focused clinical assessment, and supportive diagnostic tools, including chest computed tomography (CT) and, in selected cases, bronchoscopy.

Given the expanding use of ICIs beyond non-small cell lung cancer (NSCLC) and the pivotal role pulmonologists play in the recognition and management of this complication, a comprehensive review of CIP is warranted to support earlier diagnosis and improve patient outcomes.

## 2. Mechanism of Action of ICIs and Pathophysiology

### 2.1. Molecular Basis of Immune Checkpoint Inhibition

ICIs are monoclonal antibodies that bind to immune checkpoint proteins found on T cells and some tumor cells. By blocking these proteins, ICIs enhance the immune system’s ability to attack tumor cells. The main checkpoint proteins targeted by ICIs include cytotoxic T-lymphocyte-associated antigen 4 (CTLA-4), programmed cell death protein 1 (PD-1), and its ligand (PD-L1) [4,5].

A third class of ICIs has been more recently described: antibodies targeting lymphocyte-activation gene 3 (LAG-3). These agents restore the effector function of exhausted T cells and enhance their ability to eliminate tumor cells. LAG-3 is expressed on B cells, subsets of T cells, natural killer cells, and tumor-infiltrating lymphocytes. It promotes regulatory T-cell activity by binding to major histocompatibility complex (MHC) class II molecules and inhibiting T-cell differentiation and proliferation. In addition to MHC class II, LAG-3 also interacts with other ligands such as Fibrinogen-like protein 1 (FGL-1), Galectin-3, and Liver and Lymph Node Sinusoidal Endothelial Cell Lectin (LSECtin), contributing to T-cell exhaustion and immune evasion. Anti-LAG-3 antibodies can restore T-cell activation and show synergistic activity when combined with PD-1 inhibitors [6].

**Figure 1:**
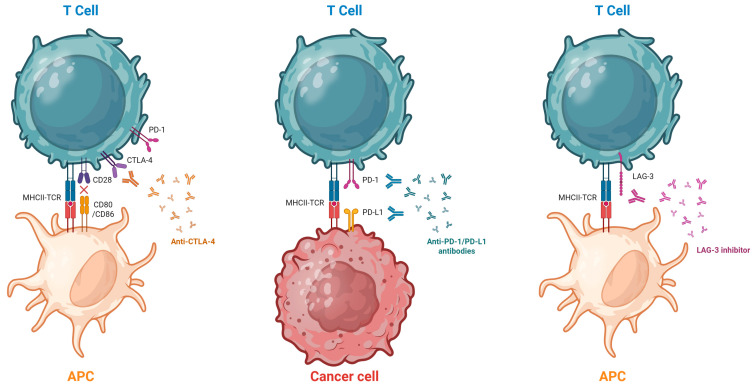
Mechanism of Action of ICIs. The T cell is activated through the interaction of its receptor (TCR) with the antigen presented by the antigen-presenting cell (APC). Full activation also requires co-stimulatory signaling via the CD28 receptor. Following activation, PD-1 and CTLA-4 are expressed on the surface of the T cell. Stimulation of the PD-1 receptor by PD-L1 expressed on the tumor cell can lead to T-cell inactivation, thereby inhibiting the antitumor immune response. Monoclonal antibodies such as anti-PD-1, anti-PD-L1, and anti-CTLA-4 block these tumor immune evasion mechanisms. Anti-LAG-3 antibodies block the interaction between LAG-3, an inhibitory receptor expressed on activated and exhausted T cells, and its major ligands, such as major histocompatibility complex class II (MHC II), restoring T cell effector function and enhancing the antitumor immune response. Created in BioRender. Hernandez, F. (2025) https://BioRender.com/tnr8t8v (accessed on 15 November 2025).

ICIs mechanisms of action are illustrated in Figure 1.

The anti-PD-1 group includes Nivolumab, Pembrolizumab, Cemiplimab, Dostarlimab, Penpulimab, Retifanlimab, Toripalimab, and Tislelizumab. The anti-PD-L1 group includes Atezolizumab, Avelumab, Cosibelimab, and Durvalumab. The anti-CTLA-4 group includes Ipilimumab and Tremelimumab. The only currently available anti-LAG-3 agent is Relatlimab, which is used in combination with Nivolumab. Several additional molecules are in development, some of which target alternative immune checkpoints [7,8,9,10,11].

### 2.2. Pathophysiology of Pneumonitis

The mechanisms underlying CIP are not yet fully understood, but several potential pathways have been proposed.

Immune dysregulation is considered a primary cause of pneumonitis, as suggested by the increased presence of activated T lymphocytes in the lungs. Some studies have reported elevated levels of T helper 1 (Th1) cells in bronchoalveolar lavage (BAL) samples of patients with CIP [12,13,14]. These cells are also elevated in certain autoimmune diseases, which may help explain the clinical similarities between autoimmune conditions and some irAEs [4,13].

When the PD-1/PD-L1 axis is blocked, differentiation to regulatory T lymphocytes is halted, which are responsible for maintaining immune tolerance, thereby improving the antitumor effect and producing immune-mediated adverse effects.

It has been described that anti-PD-L1 antibodies increase the antitumor effect of T helper 17 (Th17) lymphocytes, and that these cells, under certain circumstances, can transform into Th1 cells, secreting interferon γ, enhancing immunity and its antitumor effect [5]. The imbalance between regulatory T lymphocytes and Th17 cells is also present in several autoimmune diseases [15].

On the other hand, Th17 lymphocytes and interleukin 17 (IL-17) have paradoxically been associated with tumor generation due to their pro-inflammatory effects, as well as with toxic effects such as the development of interstitial pneumonia [16].

Some studies have associated the presence of autoantibodies with the development of irAEs. The presence of rheumatoid factor, antinuclear antibodies, antithyroglobulin, and antithyroid peroxidase antibodies were independently associated with the development of irAEs, according to a multivariate analysis [17].

Therapies targeting PD-1 cause an imbalance in regulatory T lymphocytes, generating pathological autoantibodies, such as anti-CD74, which is increased in patients with pneumonitis, stimulating the release of inflammatory mediators and suggesting a possible role of antibodies in CIP [4,5]. Based on this, it is believed that the preexistence of autoantibodies, as well as the generation of new ones, are related to the development of irAEs.

Various cytokines have been associated with the development of irAEs, with C-reactive protein (CRP), IL-6, and IL-17 being related to pneumonitis. CRP is a protein produced by the liver as an acute-phase reactant, induced by IL-6, and both have been found to be elevated in CIP. These cytokine levels increase at the time of irAE diagnosis and decrease with clinical improvement, making them useful for monitoring disease progression; however, they are not reliable for distinguishing irAEs from infection, other inflammatory conditions, or certain types of malignant neoplasms [18].

An interaction between the intestinal microbiota and immune-mediated toxicity has been proposed, with previous studies investigating the association between microbiota composition and immune-related colitis [19].

Recently, a significant study reported a distinct microbial profile in patients with CIP, compared with those with idiopathic pulmonary fibrosis (IPF) or lung cancer. This predominant microbial signature was significantly correlated with lauroylcarnitine, a metabolite shown to promote the secretion of pro-inflammatory cytokines by T cells. Such activity could lead to excessive T-lymphocyte activation, thereby contributing to the autoimmune-like responses observed in CIP [20].

## 3. Risk Factors for Pneumonitis

Understanding the risk factors for developing pneumonitis is crucial for enabling early diagnosis in at-risk patients, thereby helping to reduce the high mortality associated with this complication [21].

There are numerous studies on risk factors for irAEs, but it is difficult to unify their results, as some focus on a specific type of tumor, while others look at a particular ICI. It is also challenging to assess the relevance of the results due to the low quality of most studies. Table 1 summarizes the most relevant studies on CIP risk factors.

Risk factors for pneumonitis that have been described include: a poor ECOG performance status, smoking with a cumulative dose greater than 50 pack-years, pulmonary neoplasia [23,36], the presence of autoimmune disease [27], previous pulmonary disease (asthma, chronic obstructive pulmonary disease -COPD-, interstitial lung disease -ILD-), a history of thoracic radiotherapy, PD-1 inhibitors, and the combination of different ICIs and ICIs with chemotherapy [24].

There are several risk factors that contradict each other in different studies. For example, advanced age is proposed as a risk factor in Cho’s study [37], whereas a study from an insurance company in the United States suggests that age under 60 years is a risk factor [38], although it should be noted that this is a self-reported study, which may underestimate the incidence in older individuals. Finally, Ksienski’s study [39] states that age is not a risk factor for irAEs.

Regarding pulmonary interstitial abnormalities (ILAs), Shimoji’s study describes them as a risk factor for pneumonitis in various primary tumors, with an odds ratio of 6.29 (95% CI, 2.34–16.92; *p* < 0.001) [31], and a review supports this finding [30]. In contrast, Horiuchi’s study states that pulmonary interstitial abnormalities are not a risk factor for pneumonitis, though tobacco use may increase the risk [40].

As for radiotherapy, some studies describe it as a risk factor for CIP, particularly if it was for curative intent, distinguishing it from radiation pneumonitis by its extension beyond the irradiated area [21]. Other studies suggest that ICIs could cause a relapse of radiation pneumonitis and that they may be associated with CIP in up to 33% of cases, which could lead to confusion [41]. A relapse of radiation pneumonitis is defined as new findings on CT scans more than 6 months after conventional radiotherapy and more than one year after stereotactic body radiotherapy (SBRT). It is also noted that a relapse of radiation pneumonitis has been reported up to 5 years after radiotherapy. In Mi’s meta-analysis, the use of ICIs followed by radiotherapy showed a higher incidence of pneumonitis, compared to radiotherapy first or combined use [42].

Other risk factors for any irAEs that have been described include allergies, both food-related and to drugs or contrast agents [24].

## 4. Clinical Presentation

The symptoms of pneumonitis are non-specific. The most common are dyspnea and persistent cough, followed by hypoxemia—which may lead to respiratory failure—fever, and chest pain [43]. These symptoms may develop in an acute, subacute, or chronic manner [44].

Given the non-specific nature of the symptoms, differential diagnosis must be approached with caution, considering other possible etiologies such as respiratory infections, heart failure, myocarditis, tuberculosis reactivation, tumor progression, and radiation pneumonitis, among others [1,44].

Pneumonitis may also be asymptomatic in up to one-third of cases, where it is only detected as an incidental finding on CT scan. Imaging findings will be discussed in the following section.

On physical examination, patients with pneumonitis may present crackles on auscultation. Laboratory tests can reveal elevated acute-phase reactants, leukocytosis, neutrophilia, and CRP levels—although these findings are nonspecific [22].

Based on clinical severity presentation, CIP can be classified into four grades according to the terminology proposed by the Society for Immunotherapy of Cancer in 2017, which aligns with the Common Terminology Criteria for Adverse Events. These grades are Grade 1: asymptomatic; Grade 2: symptomatic; Grade 3: severe symptoms requiring oxygen therapy and Grade 4: life-threatening. A fifth grade (Grade 5) can also be added, referring to patients who die because of pneumonitis [5,45].

In initial clinical trials, the incidence of ICI-associated pneumonitis was reported at 3–5%, but real-world studies suggest it could be as high as 13–19% [32,46].

The latency period from the start of ICIs treatment to the development of CIP varies depending on the agent used. Combination therapy shortens latency, and more severe cases also tend to present earlier. The average time to symptom onset is approximately 1 to 2 months from the start of ICIs therapy. The most severe cases tend to occur within the first 100 to 200 days after the initiation of immunotherapy. Up to one-third of pneumonitis cases are reported to be grade 3 or higher [4,5].

**Figure 2:**
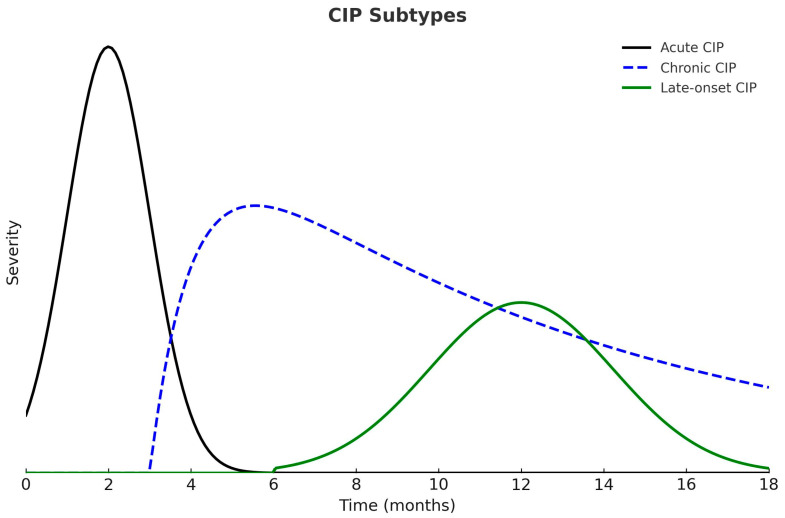
Clinical Patterns of Checkpoint Inhibitor Pneumonitis (CIP). Acute CIP develops earlier and presents with greater severity. Chronic CIP persists beyond three months after ICI discontinuation, whereas late-onset CIP appears more than three months after treatment cessation.

According to the time of onset and duration of symptoms, CIP can be classified into three clinical patterns: acute, chronic, and late-onset. Acute CIP usually develops within the first few weeks or months after initiating ICIs and, in most cases, resolves with high-dose corticosteroids followed by a six-week taper. Chronic CIP persists for more than three months after ICI discontinuation and frequently requires prolonged corticosteroid therapy to maintain symptom control. Late-onset CIP appears more than three months after discontinuation of ICIs and represents the least common clinical pattern (Figure 2) [1,47].

It is important to emphasize that mortality associated with ICI-induced pneumonitis is significant. This condition represents the irAE with the highest fatality rate, accounting for up to 35% of treatment-related deaths among affected patients [43,48].

CIP may be accompanied by other irAEs in up to 58% of cases, the most common being dermatologic manifestations, colitis, hepatitis and endocrine dysfunction [2,4]. This allows for the assumption that the various irAEs arise through similar pathophysiological mechanisms.

The development of irAEs has been associated with improved survival and treatment response in patients with advanced lung or urothelial cancer, particularly when affecting the skin or endocrine system, being of moderate severity, multisystemic in nature, with late onset, and occurring under monotherapy [49,50,51]. It can be postulated that the mechanisms through which ICIs exert antineoplastic effects also underlie the development of irAEs.

## 5. Radiology

CT is the imaging modality of choice for diagnosing CIP. It is the only method capable of detecting asymptomatic cases, which are often identified incidentally during routine surveillance scans performed for tumor response assessment [48].

**Figure 3:**
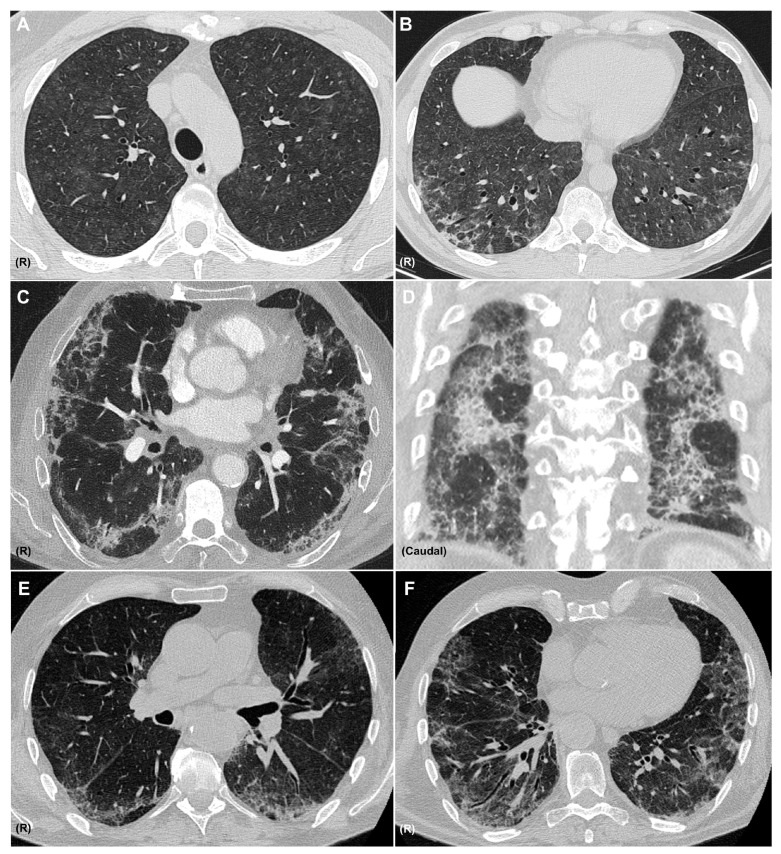
CT Patterns of Immune-Related Pneumonitis. Axial CT images showing a combined pattern, with non-fibrotic hypersensitivity pneumonitis characterized by diffuse centrilobular ground-glass nodules (**A**) and perilobular consolidative foci consistent with organizing pneumonia (**B**), in a patient with melanoma treated with nivolumab and ipilimumab. Axial (**C**) and coronal (**D**) CT images showing a combined nonspecific interstitial pneumonia (NSIP) pattern with ground-glass opacities, reticulation, and areas of subpleural sparing, along with consolidations consistent with organizing pneumonia, in a patient with pulmonary adenocarcinoma receiving pembrolizumab. Axial CT images (**E**,**F**) showing an NSIP pattern with ground-glass opacities, areas of subpleural sparing, reticulation, and traction bronchiectasis in a patient with clear cell renal carcinoma treated with nivolumab. R: right side. All CT images are fully anonymized and contain no identifiable patient information; therefore, no individual consent was necessary.

A diagnosis of pneumonitis should be considered when new pulmonary opacities appear on CT in temporal association with immunotherapy, after alternative etiologies—such as infection or tumor progression—have been excluded. It is important to recognize that a single agent may produce diverse radiologic patterns and that multiple patterns may coexist in the same patient (Figure 3). The most frequent CIP radiologic patterns are described in Table 2, in association with the sarcoid-like reaction.

The most frequently reported CT patterns in CIP include organizing pneumonia (32%) and hypersensitivity pneumonitis (16%) [24]. However, in other studies—such as the series by Naidoo et al.—ground-glass opacities (GGO) have been identified as the most common radiological finding [34].

CT imaging also plays a role in assessing the severity of pneumonitis. Severe cases are commonly associated with diffuse alveolar damage or organizing pneumonia [52]. In contrast, milder presentations typically show features consistent with hypersensitivity pneumonitis or nonspecific interstitial pneumonia (NSIP) [48].

Another ICI-induced cause of thoracic involvement is the sarcoid-like reaction, which may manifest as lymphadenopathy and perilymphatic nodules. Diagnosis requires histologic confirmation of non-necrotizing granulomas and exclusion of tumor progression. It usually carries a more favorable prognosis than CIP, as it is associated with therapeutic response to ICIs and often does not require treatment [53,54].

## 6. Diagnosis

Diagnosis of CIP requires exposure to the drug, compatible clinical features (if present), correlating CT findings, and exclusion of alternative diagnoses.

The probability scale for drug toxicity described by Naranjo et al. can be used as a diagnostic aid, bearing in mind that toxic doses are not always required to develop adverse effects with certain drugs, and that in severe cases, re-exposure to the suspected agent is not recommended [55].

A useful tool for easily accessing evidence on drug-induced pulmonary toxicity is the Pneumotox website (www.pneumotox.com), which lists the different patterns of ILD that may be associated with each drug, as well as which drugs are linked to specific ILD patterns, along with a link to the corresponding publications.

**Figure 4:**
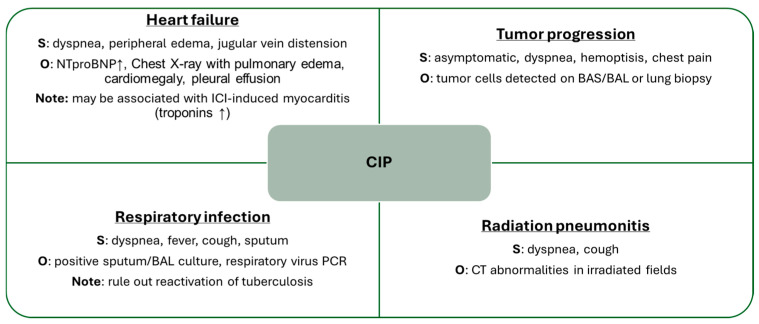
Main Differential Diagnoses of CIP. BAL: bronchoalveolar lavage, BAS: bronchial aspirate, CT: computed tomography, ICI: immune checkpoint inhibitor, NTproBNP: N-terminal pro-brain natriuretic peptide, O: observations, PCR: polymerase chain reaction, S: symptoms, ↑: elevation.

As previously mentioned, the differential diagnosis of pneumonitis is broad, and all available tools should be employed to establish this diagnosis of exclusion (Figure 4). Recommended complementary tests may include blood analysis such as N-terminal pro-B-type natriuretic peptide (NT-proBNP) and interferon-gamma release assay (IGRA), sputum or BAL cultures, cytological studies, biopsy, and, of course, chest CT imaging.

Bronchoscopy for the evaluation of CIP is recommended beginning at grade 2, although the European Society for Medical Oncology (ESMO) guideline is more permissive and considers it optional. From grade 3 onward, both the American Society of Clinical Oncology (ASCO) and Society for Immunotherapy of Cancer (SITC) guidelines recommend performing BAL. BAL is primarily used to exclude infection or neoplasia, as its findings often lack diagnostic specificity [45,56,57].

BAL lymphocytosis may serve as a supportive finding in the diagnosis of ICI-related pneumonitis in certain clinical scenarios. However, its diagnostic value remains controversial, particularly in patients with leukemia, and eosinophilia has also been reported in some cases [58].

The characteristic histopathological features of CIP biopsies usually include interstitial inflammatory infiltrates, often accompanied by eosinophilia, limited granuloma formation, and lymphocytosis [34]. Currently, lung biopsy is not routinely performed to establish the diagnosis of CIP. In most cases, the combination of clinical presentation and radiologic findings provides sufficient evidence for diagnosis, while minimizing the risks associated with invasive procedures. Biopsy is generally reserved for selected cases in which the differential diagnosis with tumor progression remains uncertain, and histopathological confirmation may significantly impact therapeutic decision-making.

Pulmonary function testing (PFT), including spirometry, diffusion capacity for carbon monoxide (DL_CO_), and occasionally the six-minute walk test, may help identify early CIP or stratify patients at risk, given that underlying lung disease is a known risk factor [59].

Most patients with lung cancer undergo PFT at the time of diagnosis to guide treatment planning. A decline in forced vital capacity (FVC) or DL_CO_, compared with baseline values, may support the early diagnosis of CIP and is also useful for monitoring disease progression. In patients with extrapulmonary malignancies, baseline PFT are less commonly available; however, in our clinical practice they are routinely employed, together with CT, for the follow-up of CIP.

It is not uncommon to face multiple challenges in establishing a diagnosis of drug-induced toxicity, including the concomitant use of multiple agents with potential pulmonary toxicity, such as Taxol agents or radiation, and the wide variability in the latency period between drug initiation and the onset of toxic effects [60].

Diagnostic yield improves when cases are discussed by a multidisciplinary team including pulmonologists, oncologists, and radiologists [43,61].

## 7. Treatment

Management of pneumonitis is based on its severity, typically graded according to clinical and radiologic criteria. Multiple oncologic societies have issued guidelines recommending similar treatment strategies [2,3,43]. Table 3 summarizes the unified recommendations from major guidelines.

Pulmonologists play a critical role in evaluating patients with CIP. Guidelines recommend specialist involvement from grade 1 onward. They contribute to imaging interpretation, perform diagnostic procedures such as bronchoscopy, and assist in differential diagnosis and therapeutic decision-making. Multidisciplinary collaboration with oncologists remains essential for optimal patient outcomes and the potential continuation of oncologic treatment [3].

The quality of evidence regarding ICI rechallenge is low; therefore, the ESMO guideline does not provide specific recommendations on this topic [56]. The other two guidelines recommend resuming ICIs after grade 1 CIP who show improvement on CT imaging, or in grade 2 cases once both symptoms and radiologic abnormalities have fully resolved [45,57]. In general, rechallenge is not recommended for grade 3 or higher pneumonitis, although the SITC guideline allows for consideration in selected grade 3 cases with complete resolution. The main risk of ICI rechallenge is recurrent pneumonitis; however, it should also be noted that CIP relapses may occur even without restarting these agents, highlighting the need for close monitoring of patients who have experienced this condition [2].

Pulmonology-led consultations dedicated to pulmonary toxicity have been established in several hospitals across Spain, highlighting the pivotal role of pulmonologists in patient care. Early referral of patients with CIP to a specialized team with expertise in managing this complication may help prevent progression to more severe stages and potentially reduce the mortality associated with this serious adverse event.

Initial treatment for pneumonitis is corticosteroid-based. If no response is observed, cases may be classified as non-responders or steroid-resistant. Non-responders show no clinical improvement, and resistant cases demonstrate partial clinical change without full resolution [47].

In a retrospective cohort including patients with various primary tumors, 14% showed no improvement with corticosteroids. Alternative immunosuppressants have been proposed but show limited efficacy and high mortality (67–100%). Most recommendations stem from low-quality evidence, lacking prospective or comparative studies. Potential options include:

- Infliximab, a tumor necrosis factor alpha (TNF-alpha) inhibitor with more data in immune-related colitis [62,63].
- Intravenous immunoglobulins (IVIG), which neutralize autoantibodies and modulate T and B cell functions, with lower infection risk [64].
- Mycophenolate mofetil, reported as a corticosteroid-resistant pneumonitis option [65,66].
- Tocilizumab and cyclophosphamide, proposed in selected cases [1,56,64].

The use of immunosuppressive therapy raises concerns regarding the potential risk of cancer recurrence, as has historically been considered in autoimmune diseases. However, current evidence remains inconclusive, and the decision to initiate such treatment should be carefully weighed against the severity of CIP [67,68].

## 8. Conclusions

CIP is one of the most feared complications of immunotherapy, whose use is rapidly expanding in oncology. Pulmonologists play a key role in identifying at-risk patients, contributing to timely diagnosis, and guiding treatment. Their expertise is particularly crucial for patients with a history of smoking, preexisting lung disease, or primary lung neoplasms—populations that warrant proactive monitoring and early screening.

Diagnosis is best approached through a multidisciplinary team, including bronchoscopy when needed to exclude infection or disease progression.

Treatment interventions go beyond severe inpatient cases, also encompassing the outpatient management of mild or chronic pneumonitis. Pulmonologists should therefore remain updated on the evolving knowledge surrounding CIP.

There are still multiple aspects of CIP that require further investigation, not only to clarify conflicting evidence, but also to gain a deeper understanding of its pathophysiology. Such insights may help explain the different clinical phenotypes and determine whether additional factors or comorbidities contribute to the development of this complication, potentially guiding more targeted therapeutic strategies.

## Figures and Tables

**Table 1:** Reported Risk Factors for Checkpoint Inhibitor Pneumonitis (CIP).

Risk Factor	Study Comments
Male sex	Reported as a risk factor in some series; female predominance noted with CTLA-4 treatment [22,23,24]
Smoking	Reported as a CIP risk factor; also risk factor for lung and renal cancer [22,23]
Autoimmune disease	Associated with higher CIP risk; auto-reactive antibodies may contribute [25,26,27]
ECOG	Poor ECOG quality of life scale score, ≥1 or ≥2 [28,29]
Lung disease	COPD and asthma described as risk factors, particularly with low FEV1 [28,29,30] ILD and ILAs reported as risk factors [23,24,28,29,30,31]
Type of malignancy	Higher frequency in NSCLC (especially squamous cell carcinoma) and renal cell carcinoma [22,32,33]
Combination of ICIs	CTLA-4 plus other ICI doubles CIP incidence; linked to more severe cases [34,35]
Radiotherapy	Prior curative-intent thoracic radiotherapy associated with CIP; differentiation from radiation pneumonitis required [21,24]

CIP: checkpoint inhibitor pneumonitis; COPD: chronic obstructive pulmonary disease; CTLA-4: cytotoxic T-lymphocyte–associated protein 4; ECOG: Eastern Cooperative Oncology Group; FEV1: forced expiratory volume in 1 s; ICIs: immune checkpoint inhibitors; ILAs: interstitial lung abnormalities; ILD: interstitial lung disease; NSCLC: non-small cell lung cancer.

**Table 2:** Radiologic Patterns of Checkpoint Inhibitor–Induced Lung Toxicity.

Pattern	CT Findings
Ground glass opacities (GGO)	Areas of increased attenuation with preserved bronchovascular marking
Organizing Pneumonia	Multifocal patchy alveolar opacities, typically with peribronchovascular and/or peripheral distribution; may show the reversed halo sign
Hypersensitivity Pneumonitis	Poorly defined centrilobular nodules, bilateral ground-glass opacities, areas of decreased attenuation and vascularity (mosaic attenuation)
Nonspecific interstitial pneumonia (NSIP)	Patchy ground-glass opacities progressing to irregular reticular opacities, architectural distortion, and traction bronchiectasis, with or without consolidation; typically bilateral, symmetric, and lower-lobe predominant
Diffuse alveolar damage	Extensive bilateral ground-glass opacities and dependent airspace consolidation in the exudative phase; traction bronchiectasis and volume loss in organizing/fibrotic phases
Sarcoid-like	Peribronchial and mediastinal lymphadenopathy either associated with perilymphatic nodules or as an isolated finding.

**Table 3:** Grading and Management of CIP.

Grade	Description	Management
1	Asymptomatic; isolated CT findings	- Monitor symptoms and repeat imaging before ICIs cycle [45,57]: If improvement/resolution → resume ICI with close monitoring [57] If progression → manage as higher grade [45] If stable → consider continuing ICIs [57] with close clinical and functional monitoring (O_2_ saturation, FVC, DL_CO_)
2	Symptomatic; limits activities of daily living	- Hold ICIs therapy [45,57] - Consider infectious disease and pulmonology consults, with bronchoscopy [45,56,57] - Consider empiric antibiotics [57] - Start prednisone 1 mg/kg/day; if improvement after 2–3 days, taper over >4 weeks [56,57] - Escalate to grade 3–4 management, if no improvement [45] - Consider ICIs rechallenge if full clinical and radiologic resolution occurs [45,57]
3	Severe symptoms; requires oxygen	- Permanently discontinue ICIs [45,56,57] - Hospitalization [45,56] - Pulmonology and infectious disease consults; bronchoscopy recommended [45,57] - Empiric antibiotics [56,57] - Initiate methylprednisolone 1–2 mg/kg/day, escalate or taper depending on response [45,57] - Rechallenge only if complete resolution and after risk-benefit assessment [45]
4	Life-threatening; may require intubation	- Permanently discontinue ICIs [45,56,57] - ICU-level care [45] - Pulmonology and infectious disease consults [45,57] - IV methylprednisolone 2 mg/kg/day [45,57] - If no improvement after 2 days: consider infliximab, cyclophosphamide, mycophenolate mofetil, or IVIG [45,57] - Taper steroids slowly over ≥2 months [45,56]
- Consider prophylaxis against Pneumocystis jirovecii in patients receiving prednisone at doses ≥20 mg/day for ≥4 weeks, treatment with calcium, vitamin D, glucose monitoring and proton pump inhibitors - Perform IGRA assay before starting TNF-alpha inhibitors (Infliximab)		

CT: computed tomography; DL_CO_: diffusing capacity of the lungs for carbon monoxide; FVC: forced vital capacity; ICIs: immune checkpoint inhibitors; ICU: intensive care unit; IGRA: interfer-on-gamma release assay; IVIG: intravenous immunoglobulin; O_2_: oxygen; TNF-α: tumor necrosis factor-alpha.

## Data Availability

The original contributions presented in this study are included in the article. Further inquiries can be directed to the corresponding author.

## Abbreviations

The following abbreviations are used in this manuscript:

APC	Antigen-presenting cell
ASCO	American Society of Clinical Oncology
BAL	Bronchoalveolar lavage
CIP	Checkpoint inhibitor pneumonitis
COPD	Chronic obstructive pulmonary disease
CRP	C-reactive protein
CT	Computed tomography
CTLA-4	Cytotoxic T-lymphocyte-associated antigen 4
DL_CO_	Diffusing capacity of the lung for carbon monoxide
ECOG	Eastern cooperative oncology group
ESMO	European Society for Medical Oncology
FEV1	Forced expiratory volume in 1 s
FVC	Forced vital capacity
GGO	Ground-glass opacities
ICIs	Immune checkpoint inhibitors
ICU	Intensive care unit
IGRA	Interferon-gamma release assay
IL-6	Interleukin 6
IL-17	Interleukin 17
ILAs	Pulmonary interstitial abnormalities
ILD	Interstitial lung disease
IPF	Idiopathic pulmonary fibrosis
irAEs	Immune-related adverse events
IV	Intravenous
IVIG	Intravenous immunoglobulin
LAG-3	Lymphocyte-activation gene 3
MHC	Major histocompatibility complex
NSCLC	Non-small cell lung cancer.
NSIP	Nonspecific interstitial pneumonia
NT-proBNP	N-terminal pro-B-type natriuretic peptide
O_2_	oxygen
PD-1	Programmed cell death protein 1
PD-L1	Programmed cell death protein 1 ligand
PFT	Pulmonary function testing
SBRT	Stereotactic body radiotherapy
SITC	Society for Immunotherapy of Cancer
TCR	T cell receptor
Th1	Lymphocytes T helper 1
Th17	Lymphocytes T helper 17
TNF-alpha	Tumor necrosis factor alpha
